# Wet-Grinding Reactivation and Frost Resistance of Waste Sludge from a Concrete Mixing Plant: A Case Study of Large-Scale Mine Construction in the Xinjie Taigemiao Mining Area

**DOI:** 10.3390/ma19183814

**Published:** 2026-09-08

**Authors:** Lin Wang, Tao Han, Mou Lv, Tingting Luo, Maolin Liu, Bing Xue, Junwei Zhang, Yongsheng Ji

**Affiliations:** 1 Shenhua Group Xinjie Energy Co., Ltd., Ordos 017200, China; 10031368@ceic.com (L.W.); 10020796@ceic.com (M.L.); 10032011@ceic.com (M.L.); 20007971@ceic.com (J.Z.); 2 State Key Laboratory of Intelligent Construction and Healthy Operation and Maintenance of Deep Underground Engineering, China University of Mining and Technology, Xuzhou 221116, China; kdltt@cumt.edu.cn (T.L.); jysbh@126.com (Y.J.); 3 Xuhai College, China University of Mining and Technology, Xuzhou 221008, China; xue2010bing2010@163.com

**Keywords:** mining construction, waste sludge, wet grinding, reactivation, compressive strength, frost resistance

## Abstract

The consumption of concrete is enormous during coal mine construction in the Xinjie Taigemiao mining area. During the concrete production process, the cleaning of concrete tank trucks and mixer equipment generates a large amount of waste sludge separated from fresh concrete. How to successfully recycle waste sludge has become a key issue that urgently needs to be solved in mine construction. In this work, a method is proposed for reactivating waste sludge into cementitious materials by wet grinding to peel off hydration products from the surface of cement particles, and the influence of aging time and wet-grinding time on the mechanical properties and frost resistance of reactivated waste sludge paste are investigated; furthermore, through testing methods such as hydration heat analysis, XRD, and SEM, the hydration and hardening mechanisms of waste sludge treated by wet grinding are analyzed. The results show that wet grinding can break the adhesion between agglomerated waste particles and peel off the hydration products from the surface of the waste particles to obtain fresh cement particles, thereby restoring the cement’s hydration activity and bonding properties. The cementitious properties of reactivated waste sludge first increase and then decrease with the increase in aging time, and they first increase and then tend to stabilize with the increase in wet-grinding time; the frost resistance of the reactivated waste sludge is improved. The optimal aging time and optimal wet-grinding time for reactivated waste sludge are 14–18 h and 10 min, respectively.

## 1. Introduction

The Xinjie Taigemiao mining area is one of China’s large-scale undeveloped integrated coalfields, a hundred-million-ton national key project run by the State Council, and a major resource succession project in the core integrated area of the China Energy Investment Corporation. The mining area is located in the central and southern parts of the Ordos Plateau in Inner Mongolia, developed by Shenhua Xinjie Energy Co., Ltd., with a total area of about 800 square kilometers and resource reserves of about 14.4 billion tons [1,2].

The plan for the mining area is to develop eight mine fields and one exploration area, with a total construction scale of 56 million tons per year. Construction of the mining area requires a huge amount of concrete. Specifically, a single mine contains three to four shafts, requiring 100,000 cubic meters of concrete construction over a 1-year period, while the eight mines will require over 1 million cubic meters of concrete in total; during the coal mine construction process, support for underground roadways will require even more concrete over a continuous period of 4 to 5 years; and construction of the industrial plaza will require approximately 800,000 cubic meters of concrete [3,4].

It is reported that for every 1 m^3^ of concrete produced, the cleaning of concrete mixer trucks and equipment produces about 0.1 tons of waste sludge, and the construction of the entire mining area will generate over 10,000 tons of waste sludge every year [5,6]. If these abandoned sludge wastes are not properly treated and utilized, they will not only cause environmental pollution such as waste accumulation and sewage runoff but also lead to resource waste and economic losses [7,8].

The waste sludge from concrete mixing plants mainly originates from production, transportation, and the use of ready-mixed concrete [9]. During the cleaning process of concrete mixer trucks and equipment, the freshly mixed concrete, under the high-pressure flushing of a water jet, is decomposed into sand and stone aggregates and waste sludge. The sand and stone aggregates can be recycled in concrete, while the waste sludge is discharged into a sedimentation tank. Waste sludge has a high water retention capacity and a loose, porous structure [10,11]. Most scholars focus on using waste sludge to press low-quality blocks and bricks. Garapati, K. P. and Shicong Kou, et al. conducted research on the preparation of partition blocks from waste sludge, and the results showed that waste sludge can improve the compressive and flexural strength of masonry blocks to a certain extent and reduce energy consumption [12,13]. However, the application range of bricks and blocks in the construction field is relatively narrow.

Some researchers have attempted to use waste sludge as a mineral admixture or aggregate to prepare concrete. Mariane Audo and He X, et al. crushed hardened waste sludge into recycled aggregate to produce concrete, but the results show that the defects of waste sludge aggregate, such as low density, high porosity, and high water absorption, lead to a significant decrease in the workability and mechanical properties of the concrete [14,15]. Chen, X.D. et al. ground waste sludge into dry powder for use as a mineral admixture, and the results showed that when the content of waste sludge exceeded 15%, the mechanical strength of the concrete decreased significantly [16]. Zhang Linglei attempted to calcine the waste sludge at high temperatures to improve its activity as a mineral admixture, but the activity of the waste sludge did not improve significantly after being calcined at 800 °C for 2 h, and the content of the waste sludge could only be increased to about 25% [17].

Existing methods for treating waste sludge do not consider the time-dependent issues during its storage process [18,19,20]. During long-term storage, the waste sludge is in prolonged contact with CO_2_ and water, causing active components of cement particles in the waste sludge to transform into calcium carbonate and loose calcium silicate hydrate, thereby losing their cementitious activity [21,22]. The inactive waste sludge used as a mineral admixture in concrete significantly reduces the mechanical strength of concrete [23,24]. In contrast, fresh waste sludge is composed of a large number of flocculates, which contain countless insufficiently hydrated cement particles. The cement particles are encapsulated by a hydration layer that restricts the release of their activity.

This study presents a novel approach to reactivating waste sludge into cementitious materials by wet grinding to peel off hydration products from the surface of cement particles. The particle size, microstructure, and main mineral composition of wet-ground waste sludge were characterized through particle size distribution (PSD), X-ray diffraction (XRD) and scanning electron microscopy (SEM) analysis in this paper. Subsequently, the wet-grinding paste of waste sludge was utilized to produce cement-based materials. Given that the performance of cement-based materials is closely correlated with the reactivation of waste sludge in a fresh state, the cementitious behavior of waste sludge within the powder matrix was examined using video electron microscopy. The effects of curing time, wet-grinding time, and dosage of waste sludge on the mechanical properties and frost resistance were investigated by compressive strength changes in mortar specimens. The reactivation mechanism of waste sludge in wet grinding was examined through SEM, XRD and MIP.

## 2. Materials and Methods

### 2.1. Materials

#### 2.1.1. Cement

In this study, P.O 42.5 ordinary Portland cement, manufactured by China United Cement Group (Beijing, China), was employed as the primary binder, which complies with the Chinese national standard GB 175-2023 [25]. The cement exhibited a measured specific surface area of 330 m^2^/kg and a density of 3.14 g/cm^3^. Its water requirement for normal consistency was determined to be 28.1%, while the fineness, expressed as the sieve residue on a 0.08 mm sieve, reached 1.02%. The oxide composition of the cement, analyzed via X-ray fluorescence (XRF) spectrometry, is summarized in Table 1.

#### 2.1.2. Waste Sludge from a Concrete Mixing Plant

The waste sludge was collected from a concrete mixing plant serving the construction of the Xinjie Taigemiao mining area. The original concrete had a strength grade of C40, with a mixture proportion of cement:water:sand:coarse aggregate:superplasticizer = 1:0.38:1.55:2.43:0.02. The cement used was from the same batch as that described in Section 2.1.1.

Such a significant amount of concrete is used for the construction of underground infrastructure. After concrete delivery, residual concrete remaining in the truck drum was washed out using high-pressure water jets, generating aggregates and waste slurry. The aggregates were recycled for use in concrete production, while the waste slurry was discharged into a sedimentation tank [26]. After sedimentation, the slurry was separated into a clarified supernatant and a settled waste sludge. The clarified water was reused for concrete mixing, while the settled waste sludge was collected as the research material in this study.

Based on process estimation, the elapsed time from fresh concrete production to waste sludge sampling was approximately 3.5 h. At this stage, the concrete was close to final setting and had lost its plasticity, while the hydration degree of the cement was still relatively low. However, the settled waste sludge had already lost its cementitious capability. The mechanisms underlying the loss of cementitious activity and the potential for its reactivation remain unclear and warrant further investigation [18].

#### 2.1.3. Other Materials

Standard sand provided by Xiamen ISO Standard Sand Co., Ltd. (Xiamen, China) was adopted as a fine aggregate to prepare mortar samples. This sand possesses an apparent density of 2.58 g/cm^3^, with particle diameters varying between 0.15 mm and 4.75 mm, and its fineness modulus is 2.9, classifying it as medium-coarse sand. Tap water was utilized during sample mixing and curing processes.

### 2.2. Preparation of Wet-Ground Waste Sludge

The cement particles contained in the waste slurry residue obtained after high-pressure washing exhibited a low degree of hydration. A large fraction of unhydrated silicate minerals (e.g., C_2_S and C_3_S) and ferrite–aluminate phases remained. These phases exhibit mineralogical characteristics similar to those of ordinary Portland cement clinkers and possess latent hydration reactivity and cementitious potential. However, due to the near-final setting of the original concrete and the destruction of the initial cementitious structure by high-pressure washing, the resulting waste slurry residue had lost its cementitious ability.

Extra water was blended into waste slurry residue to prepare a paste with a water–binder ratio of 0.5, which was then treated by wet grinding in a planetary ball mill. This wet-grinding treatment aimed to peel off the hydration product layers wrapped around cement particles, so as to recover the cementitious reactivity of these particles.

Inert agate–zirconia balls were chosen as grinding media to prevent undesired chemical reactions throughout the grinding process. The mass ratio of grinding balls to raw materials was maintained at 4:1, and the rotational speed of the planetary ball mill was kept constant at 220 rpm.

### 2.3. Experimental Program and Testing Methods

#### 2.3.1. Workability Test of Waste Sludge Paste

The untreated waste sludge and the waste sludge wet-ground for 10 min were first oven-dried. Paste specimens were then prepared at a waste sludge-to-water mass ratio of 450:225. According to the national standard of China GB/T17671-2021 [27] “Test method for expansion cement paste”, the flowability of the prepared pastes was measured to evaluate the effect of wet grinding on the workability of waste sludge paste.

#### 2.3.2. Effect of Wet-Grinding Time on the Mechanical Properties of Waste Sludge Mortar

(1) Effect of wet-grinding time 

Five groups were prepared with wet-grinding times of 0, 5, 10, 15, and 20 min. Waste sludge aged for 24 h with a replacement ratio of 50% was used to prepare mortar specimens to examine the influence of wet-grinding time on mechanical performance.

(2) Effect of aging time

A total of thirteen experimental sets were designed with aging durations set at 0, 4, 8, 12, 16, 20, 24, 28, 32, 36, 40, 44 and 48 h. Mortar samples in each group were fabricated with waste sludge at a replacement proportion of 50% and a 10 min wet-grinding period, aiming to explore how aging duration influences the mechanical performance.

(3) Waste sludge replacement ratio

Eleven groups were prepared with waste sludge replacement ratios of 0%, 10%, 20%, 30%, 40%, 50%, 60%, 70%, 80%, 90%, and 100%. Waste sludge aged for 24 h and wet-ground for 10 min was used to investigate the effect of replacement ratio on the mechanical properties of waste sludge-based mortar.

Mortar specimens incorporating oven-dried waste sludge at a mass ratio of waste sludge:water:sand = 450:225:1350 were used to investigate the effect of wet grinding on the mechanical properties of waste sludge-based cementitious materials.

Mortar samples sized 40 × 40 × 160 mm were fabricated for every experimental group in accordance with GB/T 17671-1999 (ISO standard method). The total mass of the binder was kept constant at 450 g, comprising (450 − x) g of cement and x g wet-ground waste sludge. Meanwhile, the dosage of water and standard sand remained 225 g and 1350 g, respectively. Following mold casting, the samples were stored for 24 h under the environment of 20 ± 2 °C and relative humidity above 90%. After demolding, the specimens were transferred into a standard curing environment. The compressive strength measurements were implemented when the curing ages reached 3, 7 and 28 days.

#### 2.3.3. Frost Resistance Test of Waste Sludge Concrete

Untreated and 10 min wet-ground sludge pastes aged for 8 and 16 h were selected, as well as cement, to prepare concrete specimens for freeze–thaw tests. The untreated waste sludge and the wet-ground waste sludge were first oven-dried. The concrete mix proportions are sludge paste or cement paste:sand:gravel:water-reducing agent = 1.38:1.55:2.43:0.02, and the cement-based materials were mixed with sand, gravel, and water-reducing agent to prepare 100*100*400 mm prism concrete specimens. The specimen numbers were C1, C2, C3, and C0, respectively. The concrete specimens were demolded after 24 h of standard curing in a curing room at a temperature of 20 ± 2 °C and relative humidity > 90% and then continued to be cured until 28 days of age.

The freeze–thaw cycle tests were conducted using an HC-HDK rapid freeze–thaw tester produced by Jianyan Huace Beijing Instrument and Equipment., Ltd. (Beijing, China), with a voltage of 380 V ± 10%, a maximum power of 10.5 KW, a heating power of 9 KW, and a power of 5 HP. The rapid freeze method specified in the “Test Procedure for Hydraulic Concrete” SL/T 352-2020 [28] was used to test the resistance of the concrete. Standard-cured specimens (28 days) were soaked in 20 ± 2 °C water until saturated and then placed in the freeze–thaw cycle for testing. During the freezing process, the minimum temperature at the center of the specimen was controlled at −18 ± 2 °C; during the thawing process, the maximum temperature was 5 ± 2 °C. Each freeze–thaw cycle was completed within 4 h, with the thawing time not less than 1 h. After the set freeze–thaw cycle periods (0, 30, 60, 90 cycles), the residual compressive strength of the specimens was measured. The strength loss rate of the specimens was calculated according to the test procedure to characterize the freeze–thaw resistance of the mortar, and the effect of aging time on the freeze–thaw resistance of wet-ground sludge waste mortar was studied.

#### 2.3.4. Microstructural Characterization of Reactivated Waste Sludge

To investigate the effects of wet grinding on the particle characteristics and mineralogical composition of waste sludge from concrete mixing plants, particle size distribution, morphology, and phase composition analyses were conducted on cement, untreated waste sludge, and waste sludge wet ground for 10 min.

(1) Particle size distribution (PSD)

Cement powder, raw waste sludge and waste sludge subjected to 10 min of wet grinding were dried in an oven until reaching a constant mass, followed by screening with a 200-mesh sieve. A laser particle size analyzer (Mastersizer 2000, Malvern Instruments Ltd., Malvern, UK) was adopted to test the particle size distribution of these materials, aiming to assess the particle refining effect originating from the wet-grinding treatment.

(2) Scanning electron microscopy (SEM)

Raw waste sludge and waste sludge treated by 10 min of wet grinding were dried in an oven to a constant weight, screened using a 200-mesh sieve, and sputtered with gold prior to observation. A scanning electron microscope (GAIA3, TESCAN, Brno, Czech Republic) was utilized to characterize the microscopic morphology of the specimens, with the purpose of revealing the impacts of wet grinding on particle surface microstructure.

(3) X-ray diffraction (XRD)

Mineralogical phases of the specimens were characterized via X-ray diffraction (XRD). Measurements were carried out on a Bruker D8 Advance diffractometer (Ettlingen, Germany) equipped with Cu Kα radiation, and diffraction signals were acquired within the 2θ scanning range from 5° to 70°. Cement, raw waste sludge, and waste sludge subjected to 10 min of wet grinding were tested to clarify how wet grinding alters the mineral phase assemblage of waste sludge.

#### 2.3.5. Microstructural Morphology of Cement Paste Incorporating Reactivated Waste Sludge

Specimens T24S0C50 and T24S10C50 cured for 3 days were chosen for microstructural observation owing to their relatively complete and regular morphological features. Sample surfaces were subjected to gold sputtering with an ion sputter coater, followed by morphological detection via an environmental scanning electron microscope (Quanta 250, FEI Company, Hillsboro, OR, USA). This characterization aimed to reveal how wet grinding affects the microstructure of cement matrices blended with reactivated waste sludge.

#### 2.3.6. Hydration Characteristics of Cement Paste Incorporating Reactivated Waste Sludge

(1) Heat of hydration 

The optimal aging duration, wet-grinding period and replacement proportion of waste sludge were determined according to the mechanical property data shown in Section 2.3.2. Cement pastes with a water–binder ratio of 0.5 were fabricated with wet-ground waste sludge, raw untreated waste sludge, and neat cement serving as the reference group. An automatic isothermal calorimeter (YT12959-16, Wuhan Yite Instrument Co., Wuhan, China) was adopted to continuously monitor hydration heat release over 72 h following GB/T 12959-2024 [29]. This measurement was intended to clarify the impacts of wet grinding on the hydration reaction of cement incorporated with waste sludge.

(2) XRD analysis

Following the identical screening criteria illustrated in Section 2.3.2, XRD phase analysis was implemented on cement paste samples cured for 3 days, including T24S10C50 incorporating wet-ground waste sludge, T24S0C50 containing raw waste sludge, and neat cement paste. The testing equipment adopted was a Bruker D8 ADVANCE X-ray diffractometer (Bruker, Ettlingen, Germany) equipped with Cu Kα radiation. Data collection covered a 2θ scanning scope of 5–65°, with a scanning speed of 2°/min and a step interval of 0.02°.

## 3. Results and Discussion

### 3.1. Effect of Wet Grinding on the Workability of Waste Sludge Paste

**Figure 1:**
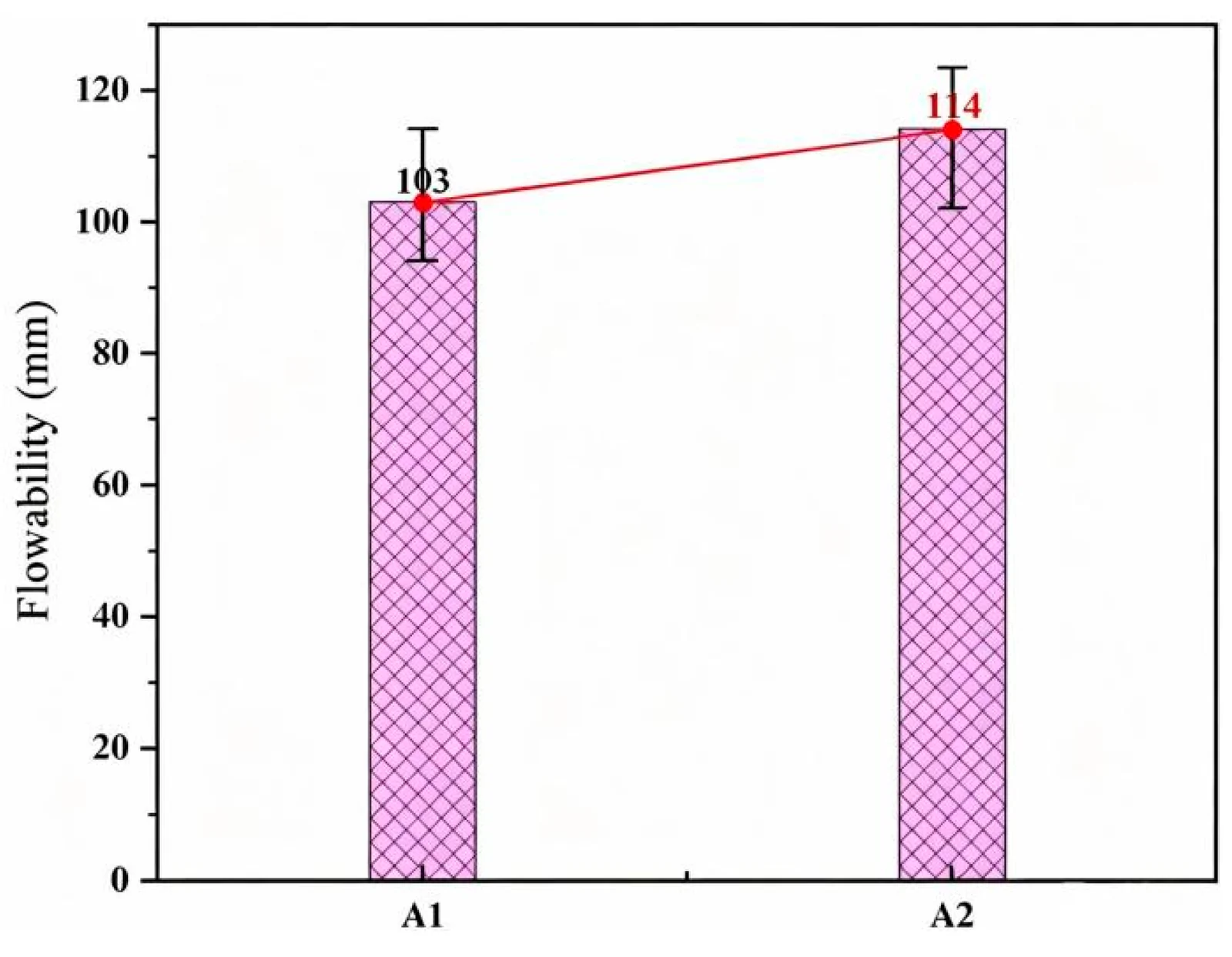
Comparison of flowability of waste sludge paste.

The flowability results of the waste sludge paste are presented in Figure 1. The flow spread of the untreated waste sludge paste (A1) was 103 mm, whereas that of the waste sludge paste subjected to wet grinding (A2) increased to 114 mm, corresponding to an improvement of approximately 10.7%. These results indicate that wet-grinding treatment can significantly enhance the workability of waste sludge paste.

This improvement is primarily attributed to the refinement of particles and deagglomeration that occur during the wet-grinding process. The hydration product shells covering the surfaces of the waste sludge particles were disrupted, and agglomerated structures were dispersed, resulting in an increased proportion of free water in the paste. Consequently, internal friction was reduced, leading to enhanced flowability.

### 3.2. Effect of Wet Grinding on the Mechanical Properties of Waste Sludge Mortar

#### 3.2.1. Effect of Wet-Grinding Time

**Figure 2:**
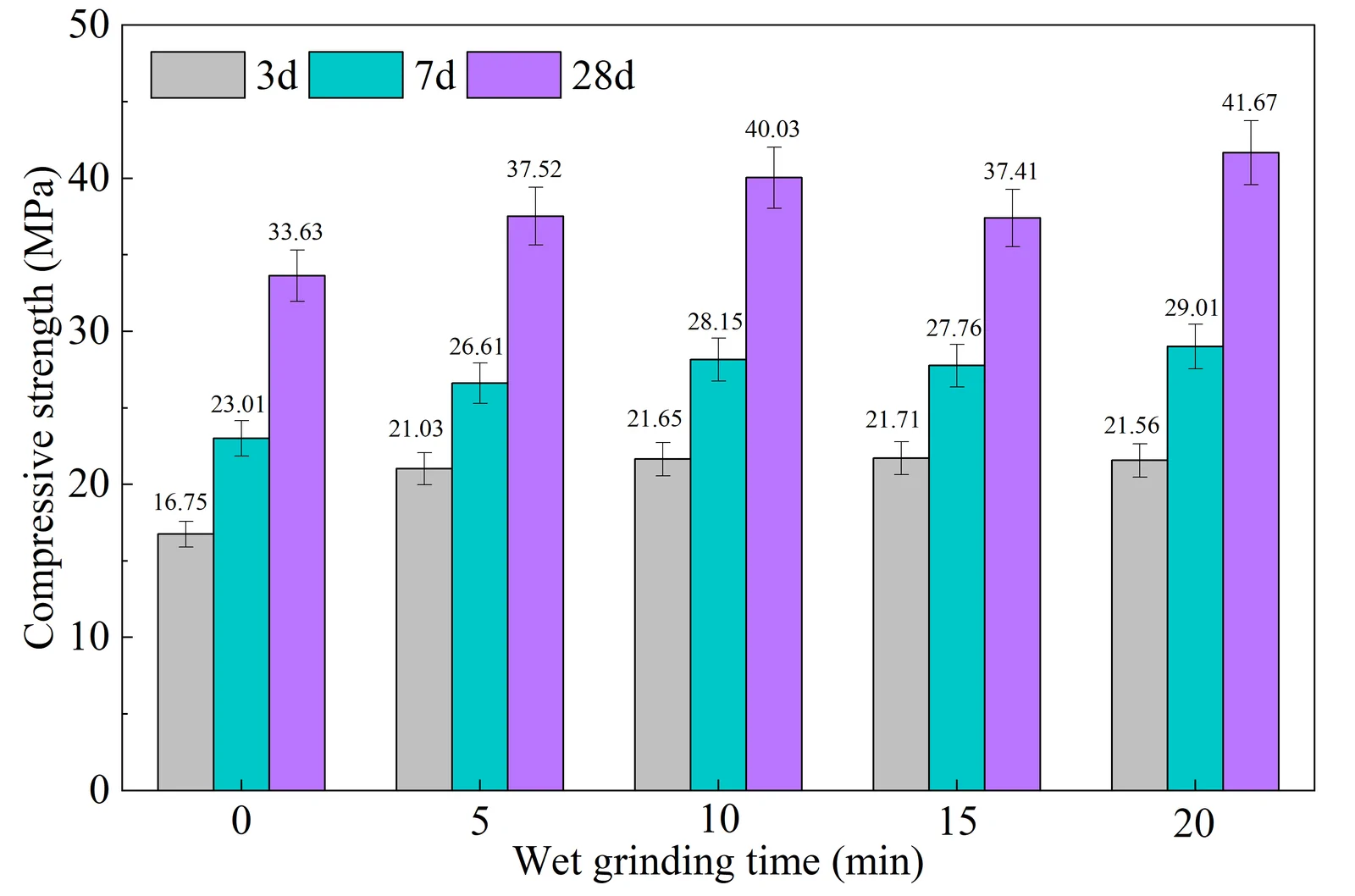
Effect of wet-grinding time on compressive strength of waste sludge–cement mortar.

Figure 2 reveals how wet-grinding duration affects the compressive strength of mortar incorporating waste sludge. For mortar mixed with raw waste sludge, compressive strengths measured at curing ages of 3, 7 and 28 days were 16.75 MPa, 23.01 MPa and 33.63 MPa, respectively. As wet-grinding duration was prolonged, the compressive strength grew substantially and followed a tendency of early growth before gradually stabilizing.

When the wet-grinding duration lasted 10 min, the compressive strengths achieved 21.65 MPa (3 d), 28.13 MPa (7 d) and 40.03 MPa (28 d). Compared with samples containing untreated waste sludge, the strength grew by 29.3%, 22.3% and 19.0%. Prolonging the grinding time further to 15 min and 20 min failed to bring about obvious additional strength promotion.

These results suggest that an appropriate wet-grinding time effectively removes the hydration product shells and exposes unhydrated cores, whereas excessive grinding provides limited further enhancement of cementitious performance.

#### 3.2.2. Effect of Aging Time

**Figure 3:**
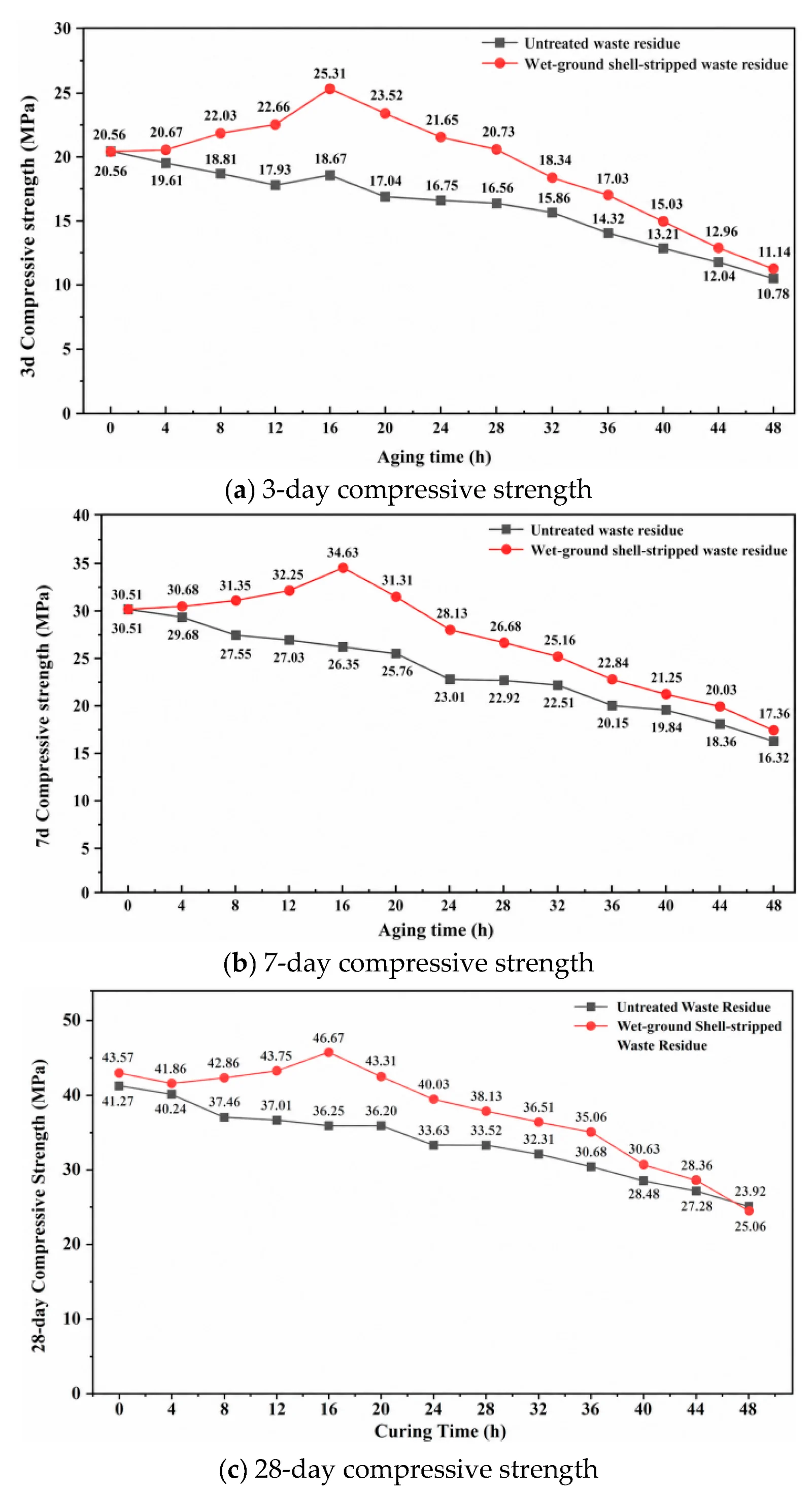
Effect of aging time on compressive strength of waste sludge mortar.

Figure 3 demonstrates how aging duration affects the compressive strength of waste sludge mortar. For mixtures incorporating raw waste sludge, compressive strength at every curing age displayed a persistent declining trend as aging time was prolonged. The mortar prepared with fresh (unaged) waste sludge achieved compressive strengths of 20.56 MPa, 30.51 MPa and 43.57 MPa after 3, 7 and 28 days of curing. Once the aging duration was extended to 48 h, these strength values dropped to 10.76 MPa, 16.32 MPa and 23.72 MPa accordingly.

In contrast, waste sludge subjected to wet grinding exhibited a distinctly different trend. At short aging times, the compressive strength was comparable to that of untreated waste sludge. Compressive strength first rises and then declines as the aging duration is extended. The peak compressive strengths of 25.31 MPa, 34.63 MPa and 46.67 MPa at curing ages of 3, 7 and 28 days were obtained at an aging duration of 16 h. These values correspond to strength enhancements of 35.6%, 31.4% and 28.7% relative to the mortar containing untreated waste sludge.

When the aging time was further extended to 48 h, the strengthening effect of wet grinding diminished significantly, and the compressive strength approached that of untreated waste sludge. This indicates that wet grinding is highly effective for short-term aged waste sludge but has limited activation potential for long-term aged materials.

#### 3.2.3. Effect of Waste Sludge Replacement Ratio

**Figure 4:**
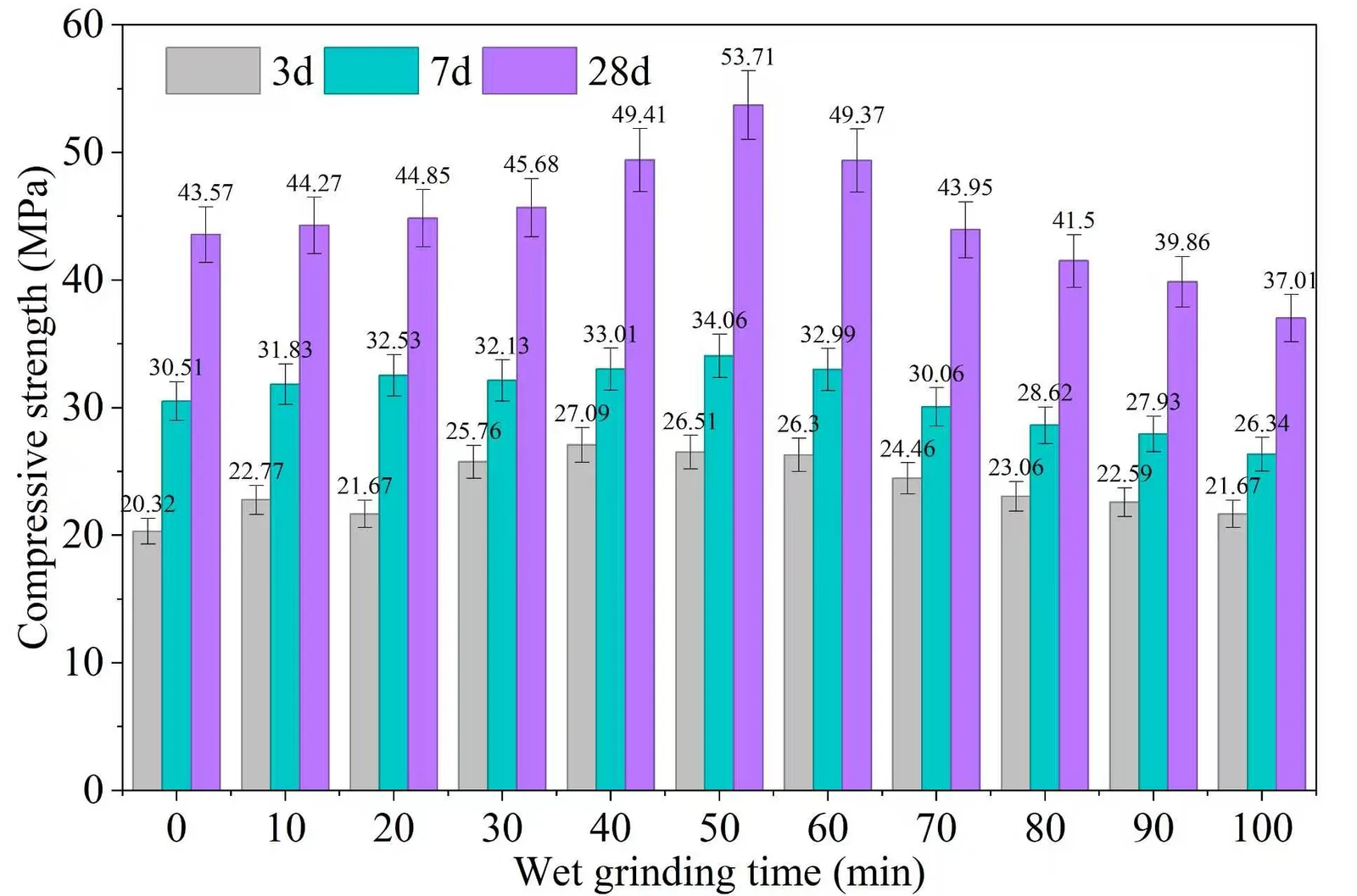
Effect of waste sludge replacement ratio on compressive strength of waste sludge mortar.

Figure 4 illustrates the compressive strength evolution of waste sludge–cement mortar as a function of waste sludge replacement ratio. The control cement mortar obtained compressive strengths of 20.32 MPa, 30.51 MPa and 43.57 MPa after 3, 7 and 28 days of curing. As cement was progressively substituted by wet-ground shell-stripped waste sludge, the mortar compressive strength displayed a trend of initial growth followed by a decline.

The peak compressive strengths of 26.51 MPa (3 d), 34.06 MPa (7 d) and 53.71 MPa (28 d) were attained at a replacement proportion of 50%. These values are 30.5%, 11.6% and 23.3% higher than those of the control mortar, respectively. This finding reveals that a suitable dosage of reactivated waste sludge can actively take part in hydration reactions and facilitate the development of mechanical strength.

Notably, after wet-grinding treatment, the mortar prepared with 100% reactivated waste sludge as the sole cementitious material exhibited compressive strengths at all curing ages comparable to those of the reference cement mortar. This result demonstrates that the reactivated waste sludge possesses sufficient cementitious activity to partially or even completely replace cement in mortar systems under appropriate processing conditions.

### 3.3. Effect of Wet Grinding on the Frost Resistance of Waste Sludge Concrete

**Figure 5:**
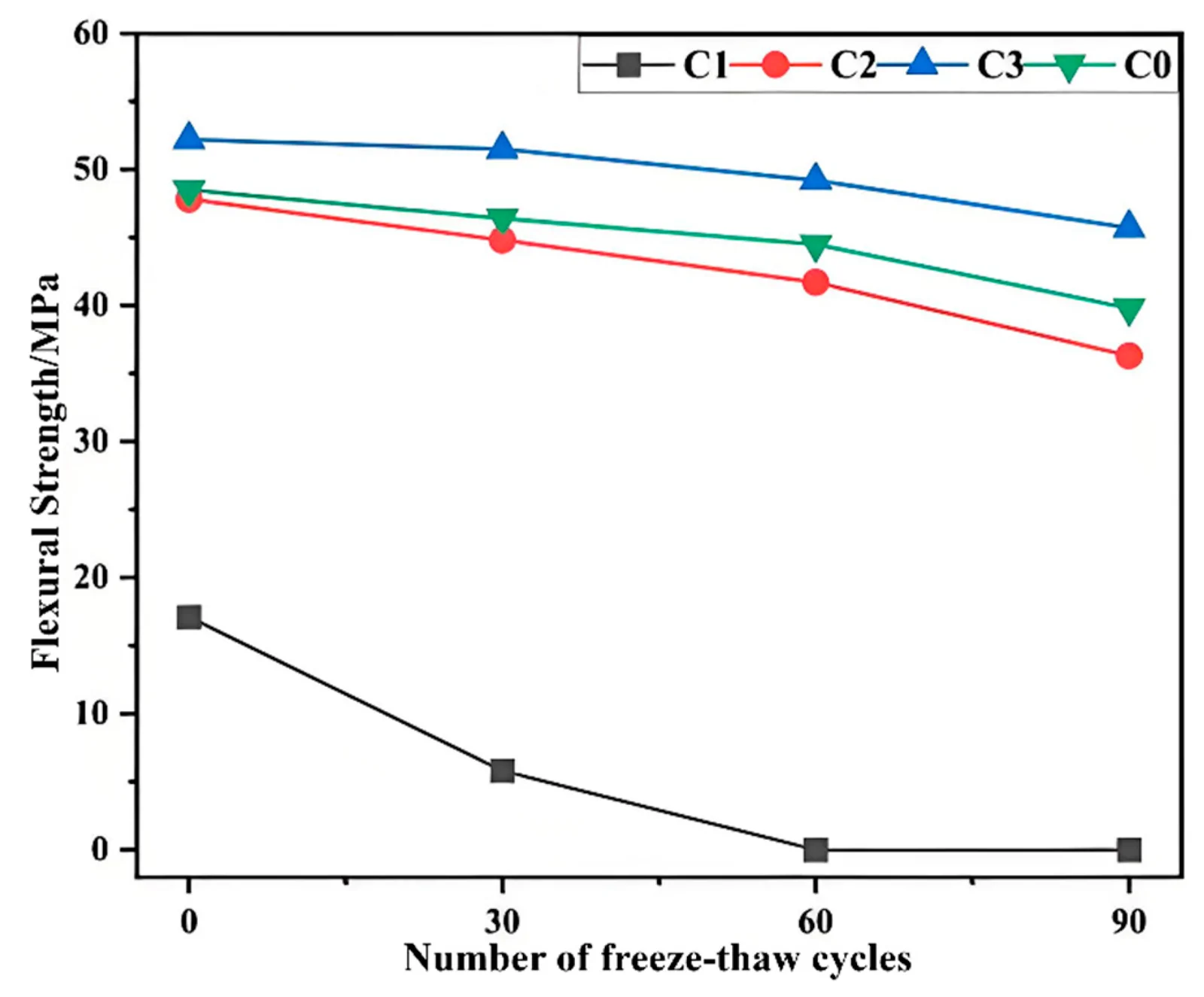
The variation in residual compressive strength with the number of freeze–thaw cycles.

The variation in the residual compressive strength of specimens in each group with the number of freeze–thaw cycles is shown in Figure 5, and the compressive strength loss rate of specimens in each group calculated from this is shown in Table 2. It can be seen from the figure and table that the cementitious properties of the untreated waste sludge are poor, with a low initial compressive strength and very poor frost resistance, and its compressive strength loss rate reaches 66.1% after 30 freeze–thaw cycles. Wet grinding can significantly improve the cementitious properties of waste sludge and the frost resistance of its concrete. The initial compressive strength of 10 min wet-ground sludge pastes aged for 8 h increased to 47.8 MPa, and after 30, 60, and 90 freeze–thaw cycles, the residual compressive strengths reached 53.9, 41.7, and 36.3 MPa, respectively, and the compressive strength loss rates decreased to 6.3%, 12.8%, and 24.1%, respectively, which is close to the level of pure cement-based concrete. The initial compressive strength of 10 min wet-ground sludge pastes aged for 16 h further increased to 52.2 MPa, and after 30, 60, and 90 freeze–thaw cycles, the compressive strength loss rates further decreased to 1.3%, 5.7%, and 125%, respectively, and its frost resistance is significantly better than that of pure cement-based concrete.

## 4. Microstructural Analysis

### 4.1. Microstructure and Mineral Composition of Reactivated Waste Sludge

#### 4.1.1. Particle Size Distribution of Wet-Grinding Waste Sludge

**Figure 6:**
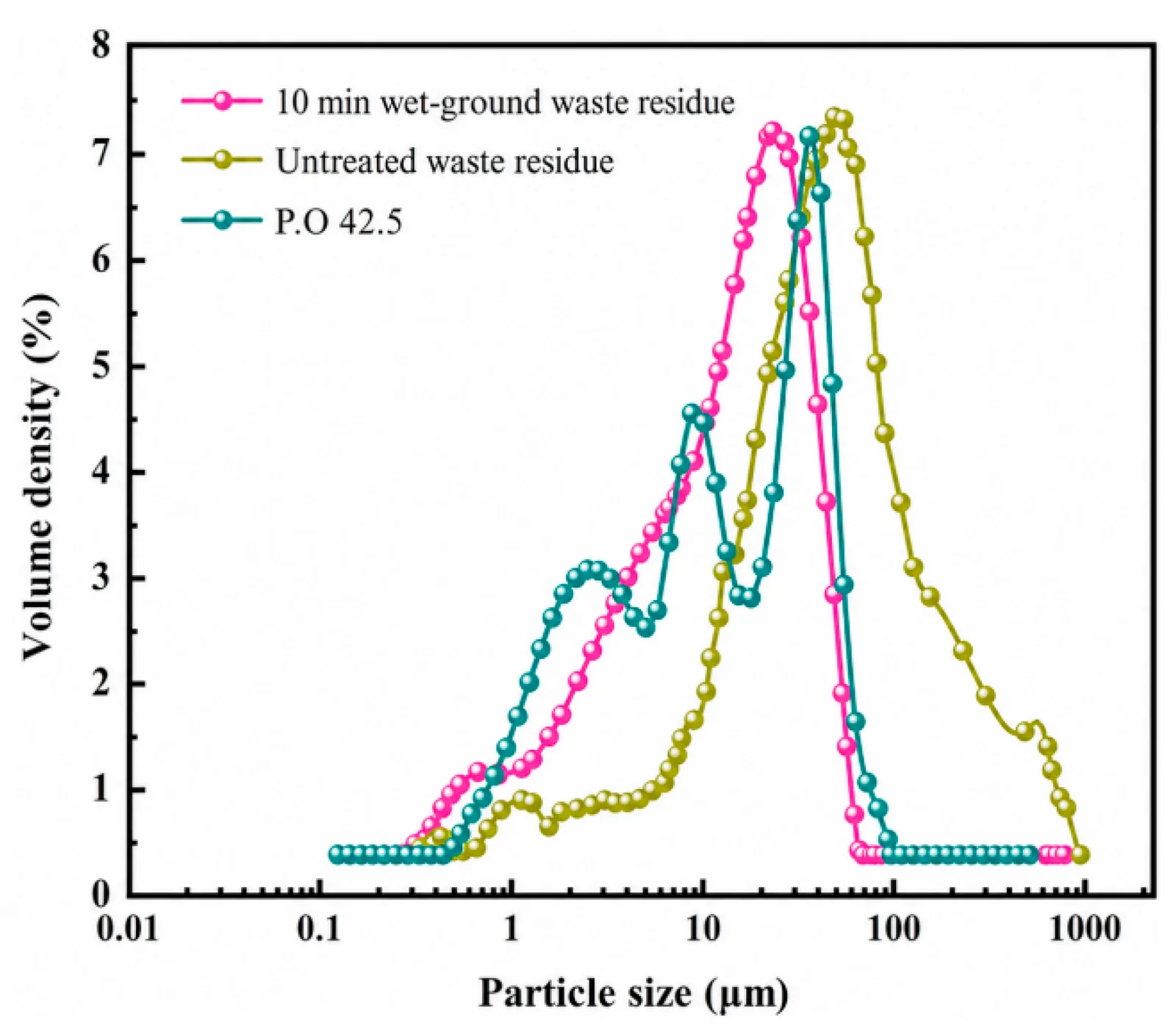
Particle size distribution of reactivated waste sludge after wet grinding.

The particle size distribution of wet-ground reactivated waste sludge is shown in Figure 6. It can be from the figure that the particle size of ordinary cement particles is mainly distributed between 1–10 μm and 20–60 μm, with a large proportion of particles having a particle size of about 30 μm. The particle size of the untreated waste is mainly concentrated between 20–70 μm, with a low content of 1–10 μm particles and a relatively high proportion of particles around 50 μm, resulting in an coarser particle size. This is because the particles in the waste sludge from the batching plant are tightly bonded to each other, leading to a larger particle size. After-grinding and de-coating treatment, the particle size distribution of the waste changes significantly, mainly concentrating between 10–40 μm, with the common particle size being 22 μm. Compared with ordinary cement particles and untreated waste particles, the particle size is more refined. This change can be attributed the fact that during the wet-grinding and de-coating process, the bonded particles in the waste sludge from the batching plant are broken, and the hydration on the surface of the waste are stripped off, thereby generating more fine hydration product particles. In addition, the wet-grinding and de-coating treatment also exposes encapsulated unhydrated cement particles, allowing them to recover their cementitious properties.

#### 4.1.2. Micro-Morphology of Reactivated Waste Sludge After Wet Grinding

**Figure 7:**
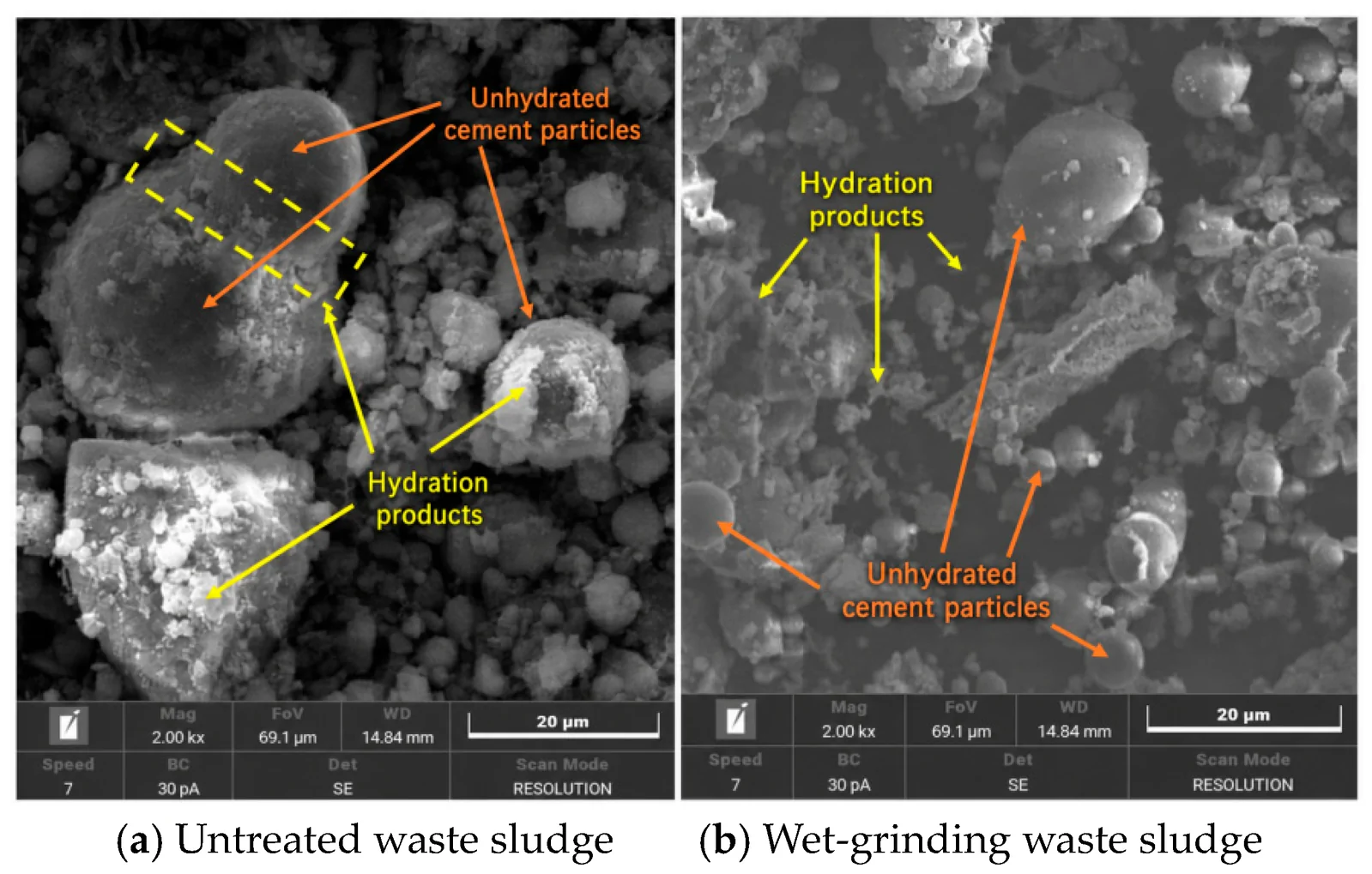
SEM images of waste sludge particles after wet grinding (×2000).

SEM images of the waste sludge before and after wet grinding are presented in Figure 7. As shown in Figure 7a, the untreated waste sludge mainly exhibits two typical morphologies. First, the particles are bonded together by hydration products to form large agglomerates with loose structures and high specific surface areas, which contribute to the high water absorption of the waste sludge. Second, individual particles exhibit rough surfaces and irregular shapes and are encapsulated by hydration products, which limits their further participation in hydration reactions.

After wet-grinding treatment (Figure 7b), the agglomeration between particles is significantly weakened. The particles tend to exhibit more regular morphologies with relatively smooth surfaces and near-spherical shapes, and a large number of fine particles are uniformly attached to the particle surfaces. These observations indicate that the wet-grinding process effectively disrupts the bonding structure between waste sludge particles and improves particle morphology, thereby providing favorable conditions for the reactivation of cementitious performance.

#### 4.1.3. Mineral Composition of Reactivated Waste Sludge After Wet Grinding

**Figure 8:**
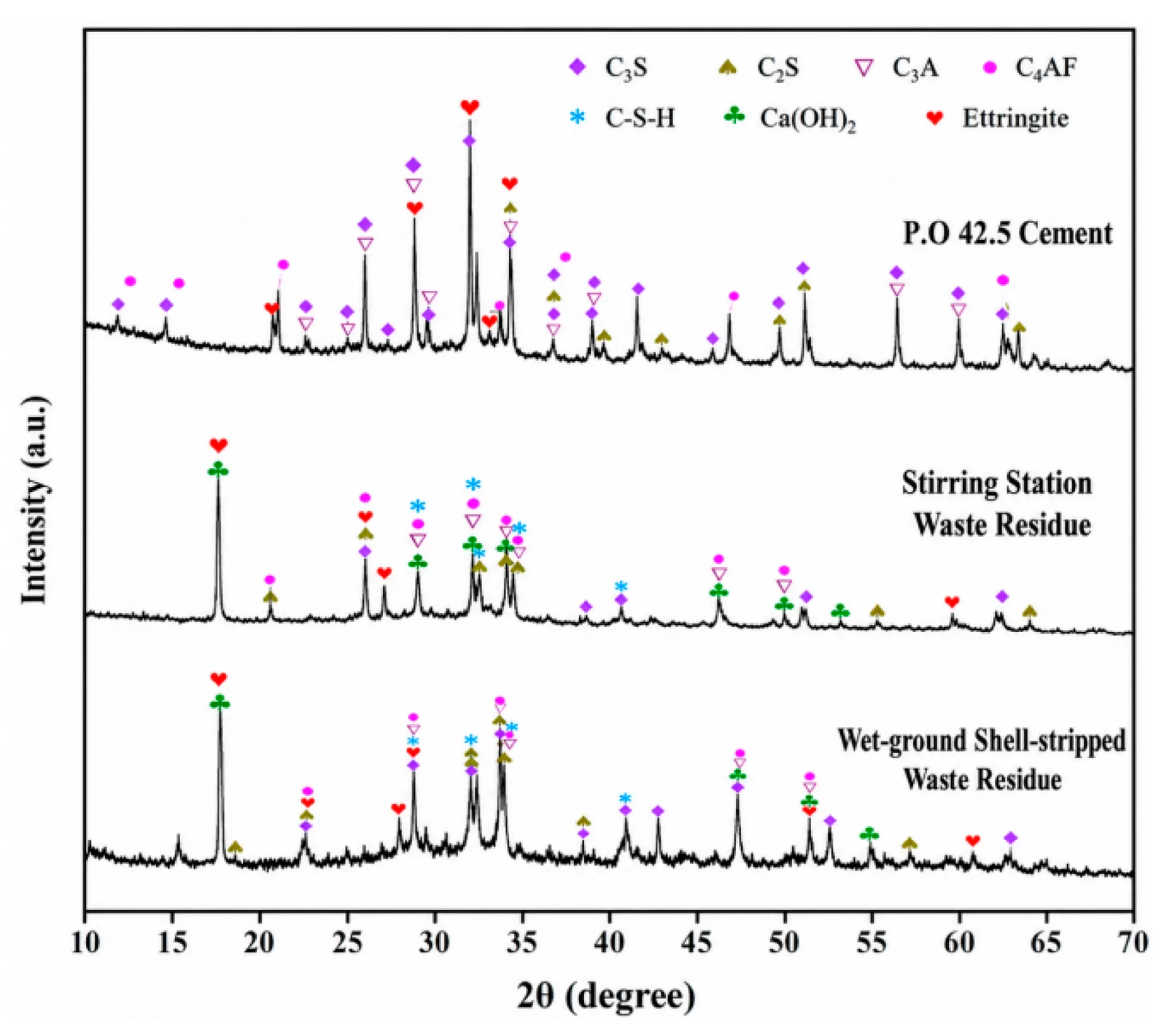
XRD patterns of waste sludge after wet grinding.

The XRD patterns of the waste sludge before and after wet-grinding treatment are shown in Figure 8. The untreated waste sludge mainly consists of C_3_S, C_2_S, C_3_A, and C_4_AF, as well as hydration products such as C–S–H and Ca(OH)_2_. Compared with ordinary Portland cement, the untreated waste sludge contains a higher amount of hydration products, while the diffraction peak intensities of clinker minerals such as C_3_S and C_2_S are significantly reduced, indicating that a considerable proportion of cement particles has already undergone hydration.

After wet-grinding treatment, no obvious change in mineral phase composition is observed. However, the diffraction peak intensities of C_3_S and C_2_S are significantly enhanced. This indicates that the hydration product layers covering the particle surfaces are effectively stripped off by the wet-grinding process, leading to the re-exposure of encapsulated unhydrated cement clinker minerals and consequently enhancing the reactivated cementitious activity of the waste sludge.

### 4.2. Microstructure of Cement Paste Incorporating Reactivated Waste Sludge

**Figure 9:**
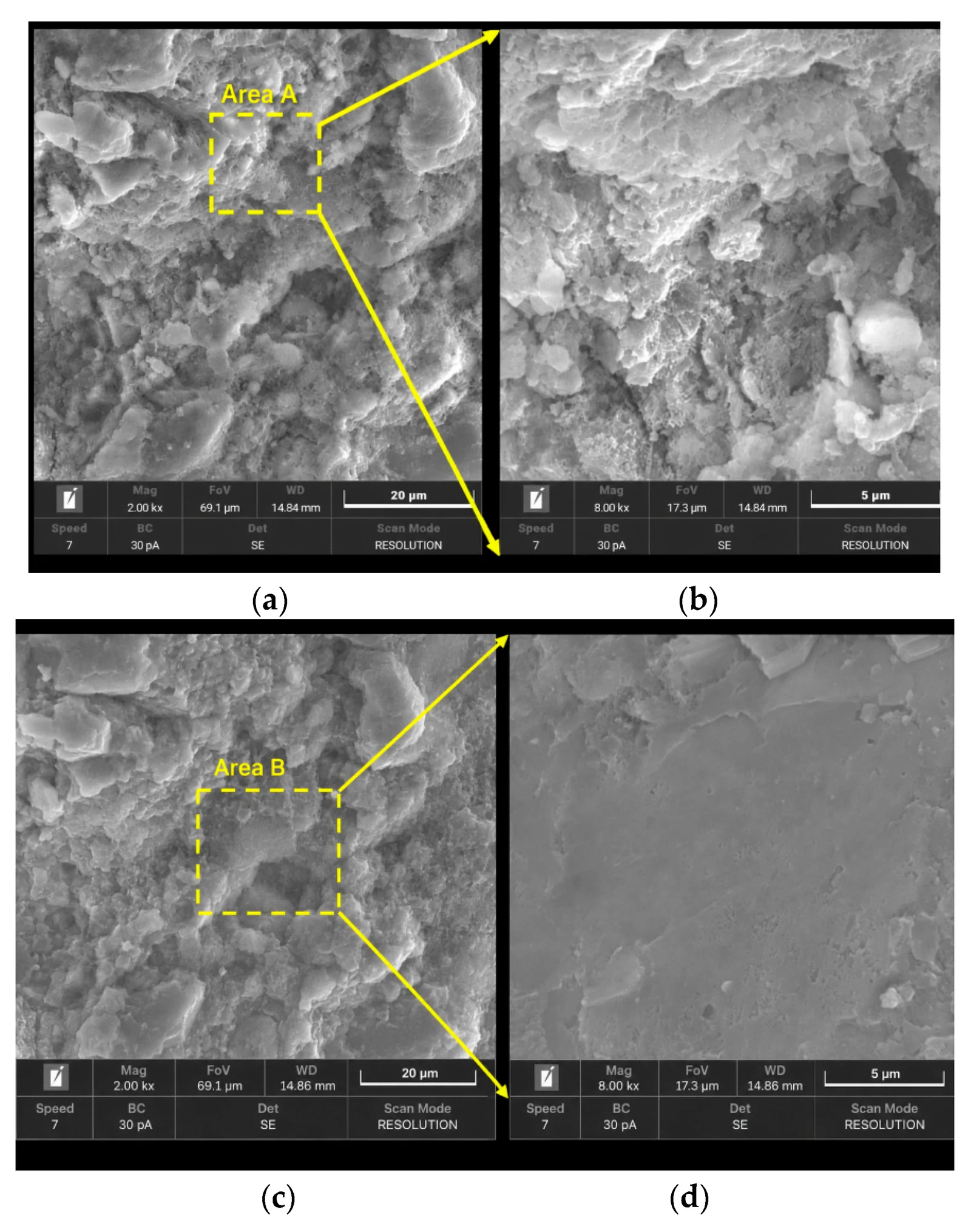
Microstructure of hardened cement paste incorporating reactivated waste sludge after wet grinding. (**a**) Cement paste with untreated waste sludge (×2000). (**b**) Magnification of region A (×8000). (**c**) Cement paste with wet-grinding waste sludge (×2000). (**d**) Magnification of region B (×8000).

SEM images of the hardened cement paste incorporating wet-ground and shell-stripped waste sludge are shown in Figure 9. As illustrated in Figure 9a, a large amount of hydration products, including amorphous C–S–H gel, Ca(OH)_2_, and ettringite, are generated within the hardened paste containing untreated waste sludge. These hydration products are interwoven, but the overall microstructure remains relatively loose with a large number of pores. The magnified image of region A (Figure 9b) further reveals that the paste surface is rough and porous, and the distribution of C–S–H gel is non-uniform.

In contrast, the hardened cement paste incorporating wet-grinding waste sludge (Figure 9c) exhibits a much denser microstructure, with a significant reduction in pore volume and a more uniform distribution of C–S–H gel. As shown in the magnified image of region B (Figure 9d), the internal structure is covered by a continuous and dense amorphous gel. This improvement in microstructure is mainly attributed to the wet-grinding process, which disrupts the bonding between waste sludge particles and removes the hydration product shell from particle surfaces, allowing fine hydration product particles to be uniformly dispersed within the matrix. These fine particles not only act as nucleation sites but also serve as fillers, effectively optimizing the pore structure and enhancing the overall compactness of the cement paste.

### 4.3. Hydration Characteristics of Reactivated Waste Sludge

#### 4.3.1. Hydration Heat Evolution

(1) Heat release rate

**Figure 10:**
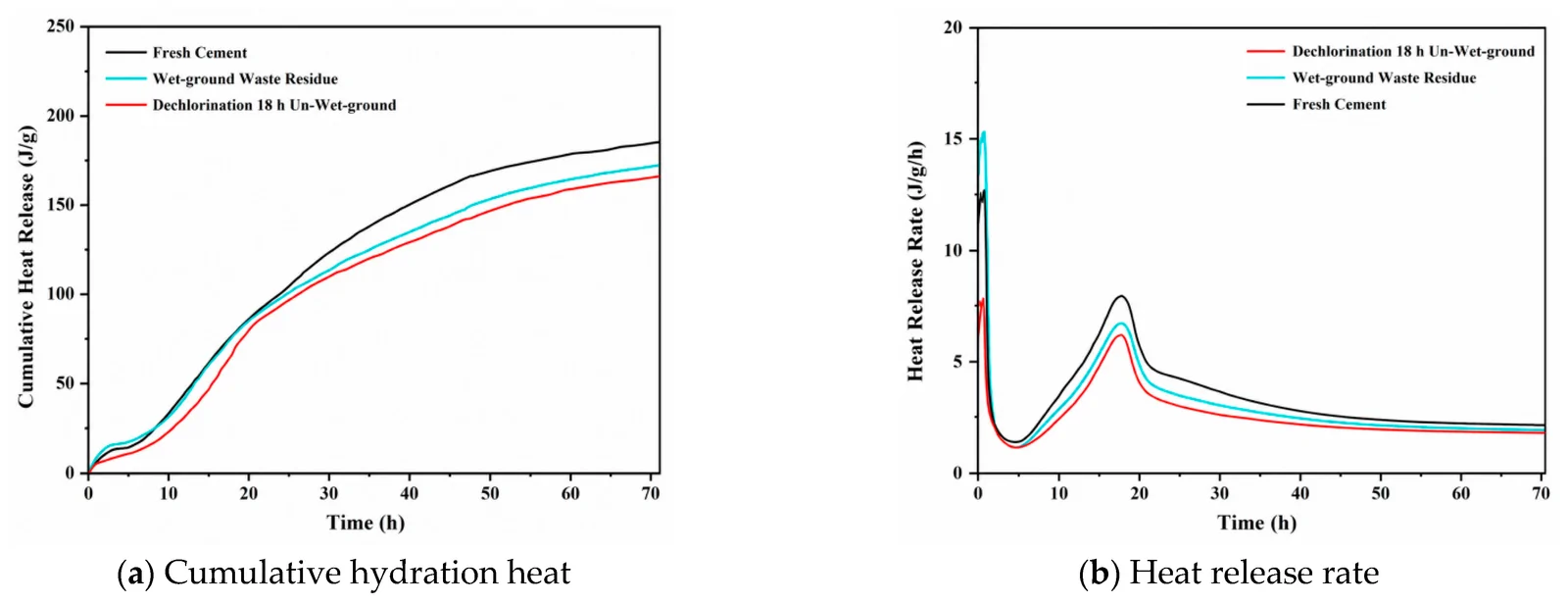
Effects of wet grinding on the hydration heat evolution of waste sludge cement.

The hydration process of cement is generally divided into five stages: the dissolution stage (I), induction stage (II), acceleration stage (III), deceleration stage (IV), and steady stage (V). The heat release rate curves of cement pastes incorporating wet-ground and shell-stripped waste sludge are shown in Figure 10a.

During the dissolution stage, cement and waste sludge particles react rapidly with water, and C_3_S and C_2_S dissolve quickly, forming the first exothermic peak. The peak values of the untreated waste sludge paste, wet-grinding waste sludge paste, and pure cement paste are 7.75, 15.96, and 13.45 J/(g·h), respectively, with the wet-grinding waste sludge exhibiting the highest heat release rate. This is mainly attributed to particle refinement and the exposure of more active surfaces induced by wet grinding, which accelerates the early dissolution process.

Upon entering the induction stage, the systems containing wet-ground and untreated waste sludge enter this stage earlier than pure cement due to their higher dissolution rates. However, during the induction stage, the heat release rate of pure cement is significantly higher than that of the waste sludge systems. This phenomenon is likely associated with the elevated ionic concentration in the liquid phase induced by the hydration of wet-ground and shell-stripped waste sludge, where excessive ions adsorb onto particle surfaces, thereby suppressing further dissolution of clinker minerals and reducing the heat release rate.

During the acceleration stage, hydration reactions are intensified, leading to the formation of the second exothermic peak. The peak values for the untreated waste sludge, wet-grinding waste sludge, and pure cement systems are 6.27, 6.66, and 7.61 J/(g·h), respectively, with pure cement exhibiting the highest peak value. This is mainly due to the partial prehydration of cement particles in the waste sludge systems, which reduces the reaction rate at later stages.

During the deceleration and steady stages, the heat release rates of all systems gradually converge. However, the onset and termination of the deceleration stage in the waste sludge systems remain slightly delayed compared with pure cement. Overall, wet grinding promotes the rapid dissolution of cement particles at the early stage, likely increasing the ionic concentration in the liquid phase. Nevertheless, the elevated ionic concentration may subsequently suppress clinker dissolution at later stages, resulting in a certain inhibitory effect on the hydration process.

(2) Cumulative heat release

Figure 10b depicts the influences of wet-grinding treatment on the cumulative hydration heat of cement blended with waste sludge. It is noticeable that within Stage I and Stage II, the cumulative heat output of cement mixed with wet-ground waste sludge is markedly higher than systems containing raw waste sludge and neat cement. Upon entering Stage III, the growth rate of heat release slows down progressively and ultimately falls below that of neat cement paste. At Stages IV and V, the cumulative hydration heat of the untreated waste sludge group, wet-ground waste sludge group and neat cement group reaches 136.68 J/g,148.16 J/g and 161.41 J/g, respectively. The wet-ground waste sludge group consistently shows a smaller cumulative heat value compared with neat cement. This phenomenon primarily arises from partial hydration of cement grains existing in waste sludge, which leads to less total available hydration heat relative to fresh cement powder.

#### 4.3.2. Hydration Products

**Figure 11:**
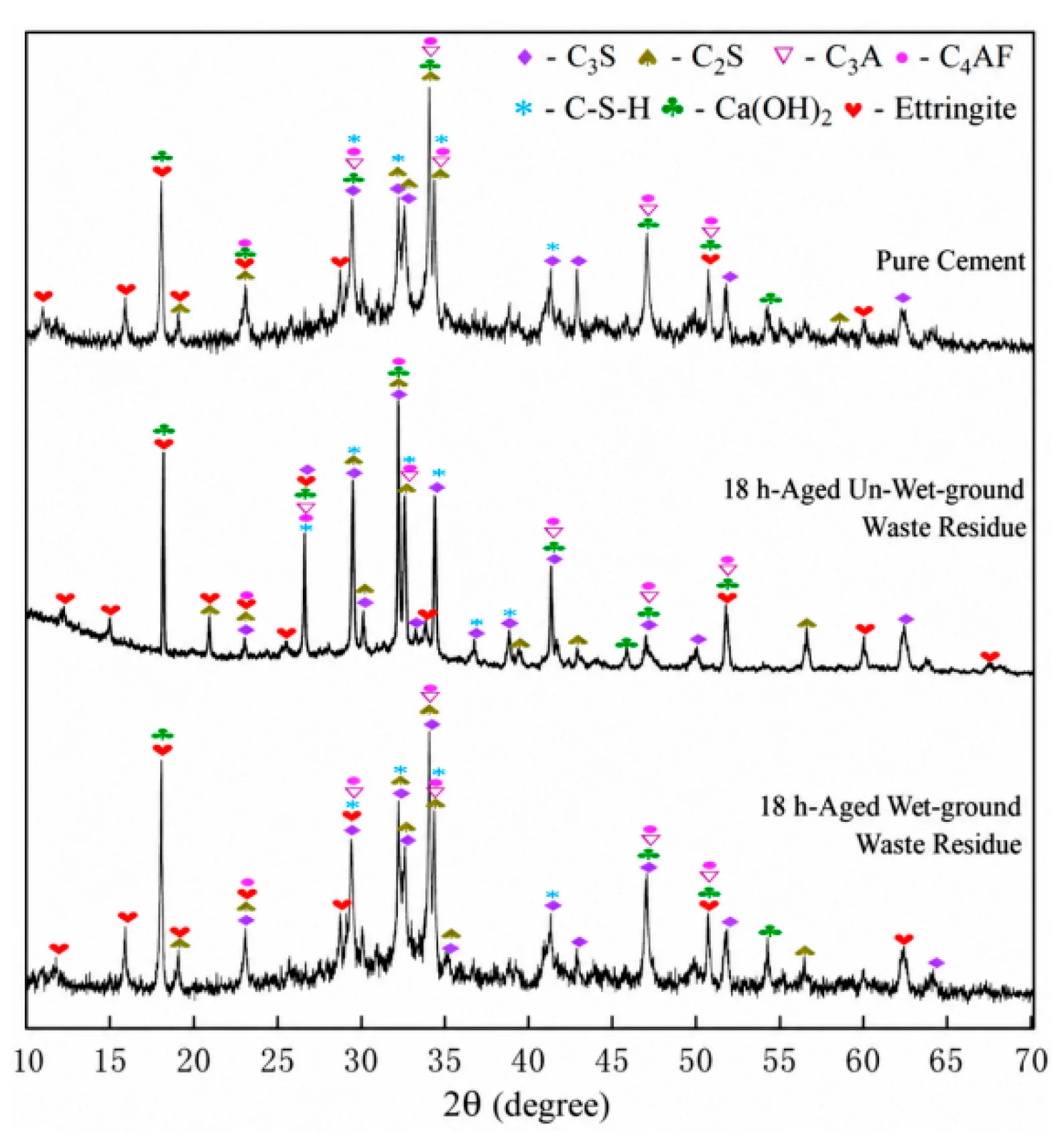
XRD patterns of cement matrices incorporating wet-ground waste sludge.

The XRD patterns of the cement matrices incorporating wet-ground and shell-stripped waste sludge are shown in Figure 11. It can be observed that the main diffraction peak positions of the three systems are essentially identical, while the peak intensities of different phases vary significantly, indicating pronounced changes in mineral phase contents after hydration. To further analyze the effects of wet-grinding reactivation treatment on hydration behavior, quantitative analysis of hydration products such as Ca(OH)_2_ was performed using the Rietveld refinement method.

The quantitative results are summarized in Table 3. Compared with the untreated waste sludge system, the contents of ettringite and Ca(OH)_2_ in the paste after wet-grinding reactivation treatment increase markedly, while the relative contents of C_3_S and C_2_S decrease. These results indicate that the wet-grinding process effectively promotes the early hydration reactions of the waste sludge system.

## 5. Mechanism Analysis

### 5.1. Cementitious Reactivation Mechanism of Wet-Grinding Waste Sludge

**Figure 12:**
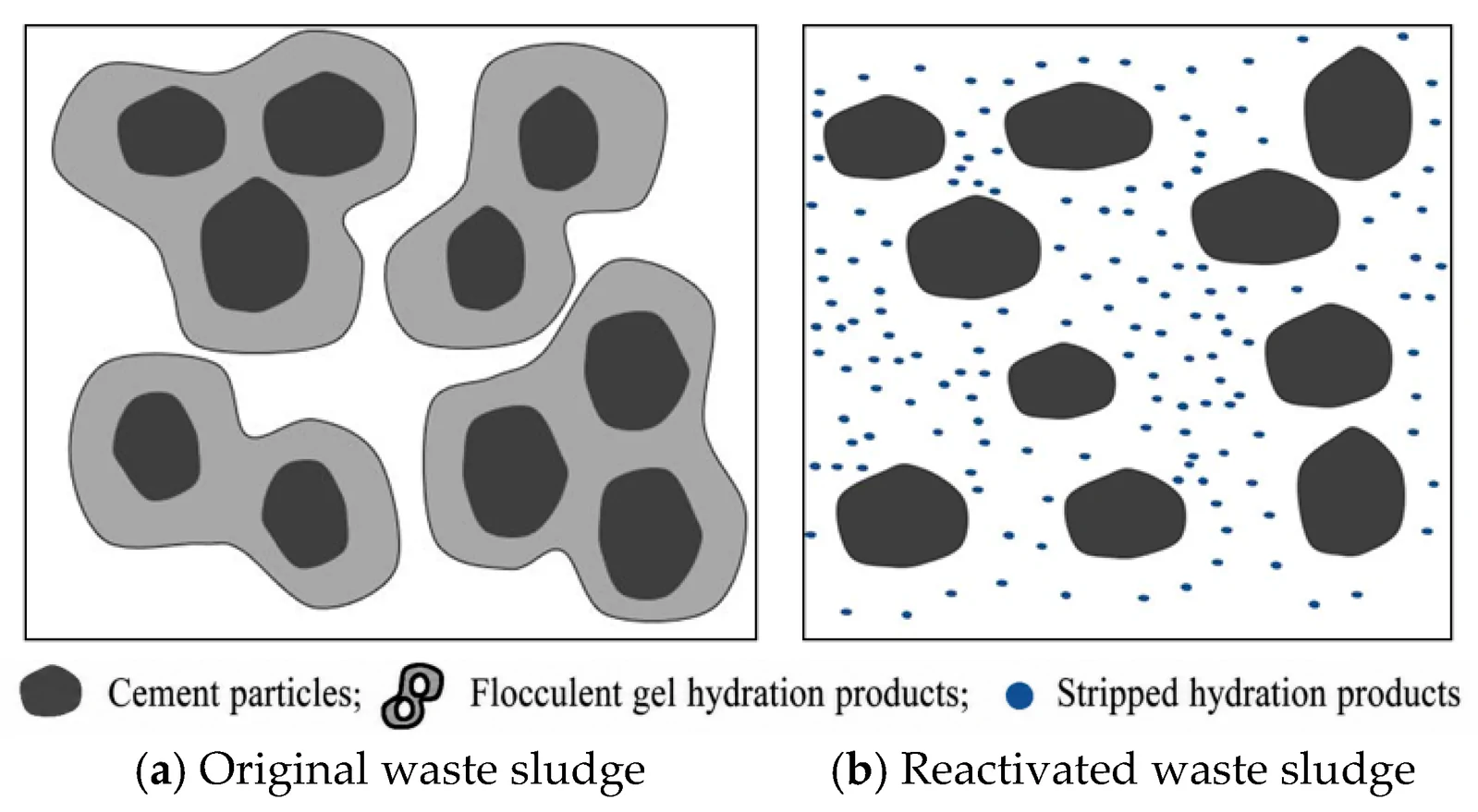
Schematic illustration of reactivation mechanism of waste sludge.

A schematic illustration of the reactivation mechanism of waste sludge through wet grinding is presented in Figure 12. In the slurry collected from concrete mixing plants, waste sludge particles are typically composed of multiple partially hydrated cement particles bonded together by hydration products formed on their outer surfaces (Figure 12a). These hydration products develop into a dense “protective shell” surrounding the particles, which not only hinders the further ingress of water into the particle interior but also isolates the unhydrated cement particles from the external hydration products [30]. As a result, the continuation of hydration reactions is significantly suppressed, leading to a pronounced reduction in or even complete loss of the overall cementitious activity of the waste sludge.

After wet-grinding treatment (Figure 12b), the protective hydration shell on the surface of the waste sludge particles is effectively stripped off under mechanical action, thereby re-exposing the unhydrated cement clinker particles to a reactive environment. Consequently, their hydration activity is restored, enabling the reactivation of the cementitious properties of waste sludge. Meanwhile, the detached hydration products are further refined into fine particles and uniformly dispersed within the cementitious system, providing abundant potential nucleation sites for subsequent hydration reactions and exerting a positive influence on the microstructural evolution of the cement matrix.

### 5.2. Hydration Pathways in Cement Paste Containing Wet-Ground Waste Sludge

#### 5.2.1. Hydration Behavior of Cement Paste Containing Untreated Waste Sludge

**Figure 13:**
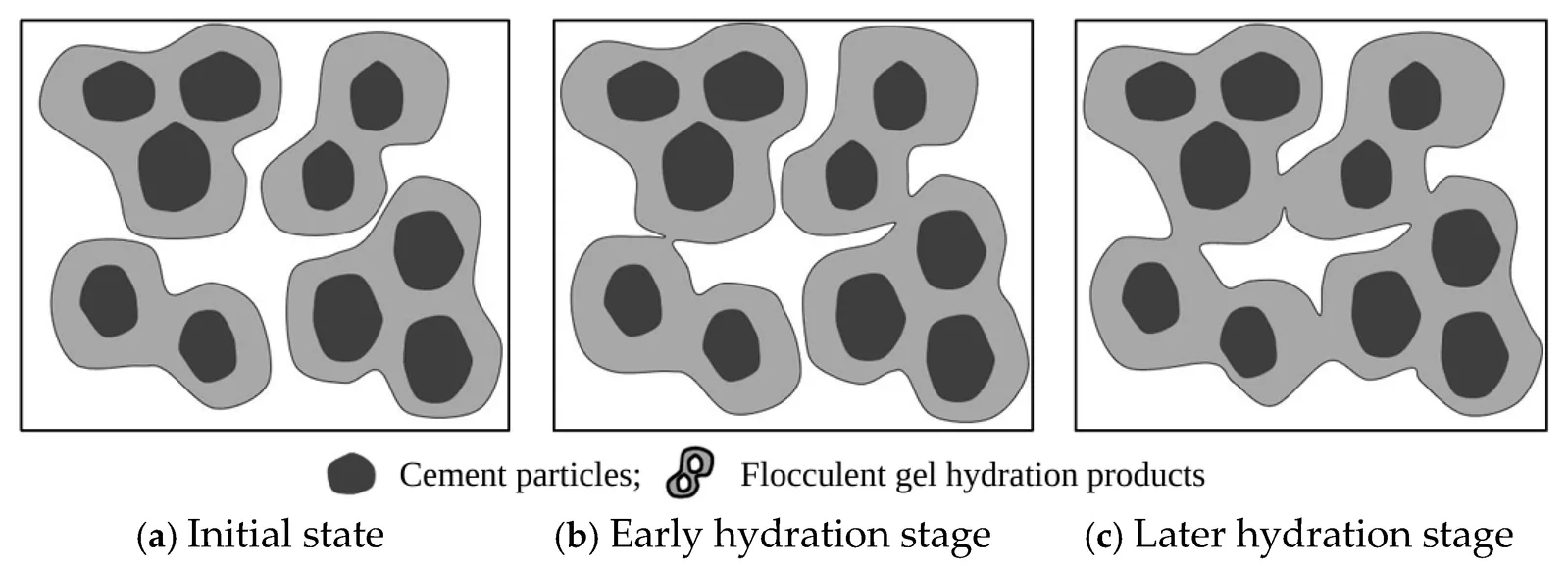
Hydration mechanism of cement paste containing untreated waste sludge.

The hydration process of cement paste containing untreated waste sludge is schematically illustrated in Figure 13. At the initial stage of hydration, waste sludge particles come into contact with water and rapidly generate a limited amount of hydration products on their surfaces, which subsequently harden into a rigid protective shell (Figure 13a). However, owing to the presence of this relatively dense hydration shell, newly formed hydration products are unable to penetrate outward or establish effective connections with neighboring particles or hydration products (Figure 13b). Consequently, only a very small number of adjacent waste sludge particles are locally bonded through thin and weak layers of newly generated hydration products [31].

As hydration proceeds slowly, the accumulation of hydration products increases, and partial interparticle connections begin to form (Figure 13c). Nevertheless, due to the weak bonding interfaces and the presence of a considerable number of interconnected pores within the paste, the resulting hardened structure remains relatively loose. This microstructural deficiency ultimately limits the macroscopic mechanical performance of the hardened paste.

#### 5.2.2. Hydration Mechanism of Cement Paste Containing Wet-Ground Waste Sludge

**Figure 14:**
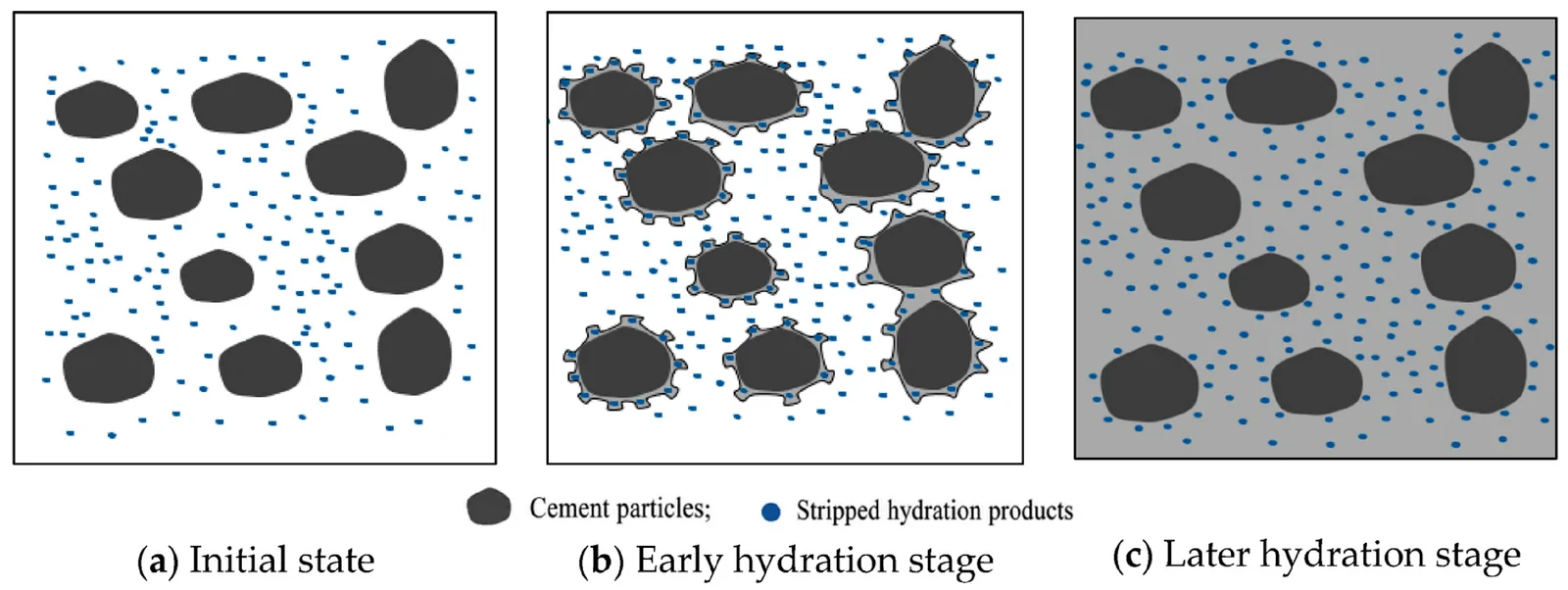
Hydration mechanism of cement paste containing reactivated waste sludge.

The hydration process of cement paste incorporating wet-ground waste sludge is illustrated in Figure 14. After wet-grinding shell removal, the hydration shell on the surface of the waste sludge particles is fragmented and refined into nanoscale particles, while the internal unhydrated cement particles are fully exposed (Figure 14a). At the early hydration stage, these exposed cement particles readily participate in hydration reactions, and the newly formed hydration products gradually envelop and interact with the dispersed nanoscale hydration particles [32,33].

These nanoscale hydration particles play a pronounced nucleation role within the system, accelerating the nucleation and growth of hydration products and enhancing the interconnection between cement particles, thereby promoting the formation of a more continuous and compact internal structure (Figure 14b). With the further progression of hydration, the synergistic effects of nucleation and pore filling by fine hydration products continuously reduce internal porosity. As a result, a dense and well-integrated hardened matrix with superior mechanical performance is ultimately formed (Figure 14c).

## 6. Conclusions

(1) The waste sludge separated from fresh concrete that has passed initial setting loses its cementitious ability due to destruction of initial cementitious structure by high-pressure flushing. Wet grinding can break the adhesion between agglomerated waste sludge particles and peel off the hydration products from the surface of cement particles in waste sludge to obtain new cement particles, thereby restoring the hydration activity and bonding performance of cement particles in waste sludge.

(2) In the wet-grinding process, the impact of the grinding balls breaks up the aggregation of waste sludge particles, releasing the free water trapped within the agglomerates, which significantly improves the working performance of the waste sludge slurry.

(3) The cementitious properties of reactivated waste sludge first increase and then decrease with the increase in aging time and first increase and then tend to stabilize with the increase in wet-grinding time; the frost resistance of the reactivated waste sludge is improved. The optimal aging time and optimal wet-grinding time for reactivated waste sludge are 14–18 h and 10 min, respectively.

(4) The hydration degree of cement particles in waste sludge increases with the aging time, causing the inherent potential cementitious properties to gradually be lost; the earlier the wet-grinding treatment is performed, the better the cementitious properties of the waste sludge are reactivated; the aging time of the waste sludge should preferably not exceed 24 h.

(5) This study demonstrates technically promising laboratory-scale reuse of sludge replacement. However, the sludge originates from one C40 concrete formulation and one mixing plant, while its composition will inevitably vary with cement type, admixtures, aggregates, wash–water ratio, production schedule, and storage conditions. The feedstock variability, scale-up of wet grinding, grinding-energy demand, and the need for validation using sludge from multiple production batches remain to be further studied.

## Figures and Tables

**Table 1:** Chemical composition of cement and waste sludge from concrete mixing plant.

Chemical Composition	SiO_2_/%	Al_2_O_3_/%	Fe_2_O_3_/%	CaO/%	SO_3_/%	MgO/%	LOI/%
Cement	22.43	5.38	3.22	65.5	2.53	2.11	0.13
Waste sludge	24.52	5.61	3.37	55.48	2.11	2.62	3.45

**Table 2:** Loss rate of compressive strength with the number of freeze–thaw cycles.

Specimens	Number of Freeze–Thaw Cycles		
	30	60	90
C1	66.1%	100%	100%
C2	6.3%	12.8%	24.1%
C3	1.3%	5.7%	12.5%
C0	4.3%	8.2%	17.9%

**Table 3:** Relative contents of crystalline hydration phases.

Number	C_3_S	C_2_S	Ca(OH)_2_	Ettringite	SiO_2_
P.O 42.5	29.1	21.3	30.9	15.9	1.8
Untreated waste sludge	43.6	34.1	3.6	14.6	3.2
Wet-ground and shell-stripped waste sludge	26.7	14.3	38.0	18.7	2.3

## Data Availability

The original contributions presented in this study are included in this article. Further inquiries can be directed to the corresponding author.
